# Correction: Musculoskeletal pain trajectories of employees working from home during the COVID-19 pandemic

**DOI:** 10.1007/s00420-022-01925-w

**Published:** 2022-10-12

**Authors:** Jodi Oakman, Subas Neupane, Saila Kyrönlahti, Clas-Håkan Nygård, Katrina Lambert

**Affiliations:** 1 Centre for Ergonomics and Human Factors, School of Psychology and Public Health, La Trobe University, Bundoora, 3086 Australia; 2 Unit of Health Sciences, Faculty of Social Sciences, Tampere University, Tampere, Finland; 3 School of Psychology and Public Health, La Trobe University, Bundoora, Australia

## Correction: International Archives of Occupational and Environmental Health 10.1007/s00420-022-01885-1

Unfortunately all authors first and family names are published incorrectly. The correct names are below:

Jodi Oakman

Subas Neupane

Saila Kyrönlahti

Clas-Håkan Nygård

Katrina Lambert

